# Loss of vesicular dopamine release precedes tauopathy in degenerative dopaminergic neurons in a *Drosophila* model expressing human tau

**DOI:** 10.1007/s00401-013-1105-x

**Published:** 2013-03-15

**Authors:** Ting-Han Wu, Yu-Ning Lu, Chia-Lung Chuang, Chia-Lin Wu, Ann-Shyn Chiang, David E. Krantz, Hui-Yun Chang

**Affiliations:** 1 Department of Medical Science, Institute of Systems Neuroscience, National Tsing Hua University, 101, Section 2 Kuang-Fu Road, Hsinchu, 30013 Taiwan; 2 Department of Life Science, Institute of Biotechnology, National Tsing Hua University, 101, Section 2 Kuang-Fu Road, Hsinchu, 30013 Taiwan; 3 Brain Research Center, National Tsing Hua University, 101, Section 2 Kuang-Fu Road, Hsinchu, 30013 Taiwan; 4 Department of Biochemistry, Graduate Institute of Biomedical Sciences, College of Medicine, Chang Gung University, Tao-Yuan, Kwei-Shan 333 Taiwan; 5 Department of Psychiatry and Biobehavioral Science, Gonda Center for Neuroscience and Genetics Research, David Geffen School of Medicine at University of California, Los Angeles, CA 90095 USA

**Keywords:** Parkinson’s disease, Dopamine, Tau, MAPT, Neuron degeneration, Tauopathy

## Abstract

While a number of genome-wide association studies have identified microtubule-associated protein tau as a strong risk factor for Parkinson’s disease (PD), little is known about the mechanism through which human tau can predispose an individual to this disease. Here, we demonstrate that expression of human wild-type tau is sufficient to disrupt the survival of dopaminergic neurons in a *Drosophila* model. Tau triggers a synaptic pathology visualized by vesicular monoamine transporter-pHGFP that precedes both the age-dependent formation of tau-containing neurofibrillary tangle-like pathology and the progressive loss of DA neurons, thereby recapitulating the pathological hallmarks of PD. Flies overexpressing tau also exhibit progressive impairments of both motor and learning behaviors. Surprisingly, contrary to common belief that hyperphosphorylated tau could aggravate toxicity, DA neuron degeneration is alleviated by expressing the modified, hyperphosphorylated tau^E14^. Together, these results show that impairment of VMAT-containing synaptic vesicle, released to synapses before overt tauopathy may be the underlying mechanism of tau-associated PD and suggest that correction or prevention of this deficit may be appropriate targets for early therapeutic intervention.

**Electronic supplementary material:**

The online version of this article (doi:10.1007/s00401-013-1105-x) contains supplementary material, which is available to authorized users.

## Introduction

Parkinson’s disease (PD) is the second most common neurodegenerative disorder, affecting 1–2 % of people over the age 60, and is characterized by clinical manifestations of bradykinesia, rigidity, resting tremor, and postural instability. These motor defects are thought to result from degeneration of pigmented dopaminergic (DA) neurons in the basal ganglia, particularly those projecting from substantia nigra pars compacta to the striatum [13, 21, 45]. Deposition of Lewy bodies or formation of Lewy neurites, composed of fibrillar aggregates of α-synuclein, are the classical pathological hallmark of PD [48]. However, tau-containing neurofibrillary tangles (NFTs) are also found in the brains of PD patients [60]. Although the inherited forms of PD are rare, genetic variation in PD susceptibility genes may play a significant role, through gene–environment interactions, in the development of sporadic PD [45].

Microtubule-associated protein tau (MAPT), leucine-rich repeat kinase 2 (LRRK2), and α-synuclein (SNCA*)* have been identified as the top three PD susceptibility genes [46]. Both SNCA and LRRK2 have been extensively studied in vitro and in vivo; however, the molecular and pathological role of MAPT in PD remains poorly understood [57]. The link between MAPT and disease, originally identified for inherited autosomal dominant frontotemporal dementia and Parkinsonism, is linked to chromosome 17 (FTDP-17). A similar association was later found in several rare atypical Parkinsonism syndromes, including progressive supranuclear palsy (PSP) and corticobasal degeneration (CB) [20, 22, 28]. More recently, a number of studies have suggested that genetic variants in MAPT can also predispose an individual to the development of sporadic and familiar forms of PD [45]. Interestingly, most mutations of MAPT associated with FTDP-17 are located in protein coding sequences for microtubule-binding repeats and splicing sites [22], whereas the mutations identified in allelic variants of MAPT associated with PD are located in upstream regulatory elements [17].

The relationship between human tau and PD is illustrated by a number of genome-wide association studies (GWAS) [16, 32, 38, 46]. The MAPT H1 haplotype is associated with an increased risk of PD [17]. In addition, individuals homozygous for MAPT H1/H1 have an increased susceptibility to develop PD compared to those bearing the heterozygous H1/H2 genotype [59]. Furthermore, some of the MAPT variants appear to increase overall Tau expression [25]. Together, these studies suggest that elevated expression of tau may increase the risk of PD via a genetically gain-of-function mechanism, similar to the duplication of the amyloid precursor proteins gene (APP gene) [43] or duplication or triplication of SNCA (encodes α-synuclein) [47]. However, unlike aberrant copy numbers of APP and SNCA, which have been clearly identified in hereditary AD and PD pedigrees, respectively, evidence for a relationship between MAPT gene dosage and either PD or tauopathies remains primarily circumstantial.

In the present study, we explored whether expression of wild-type MAPT affects DA neurons, the most vulnerable cells in PD, by using a *Drosophila* animal model. We found that expression of wild-type human tau (htau^WT^) produces the progressive degeneration of DA neurons. In addition, we observed age-dependent formation of tau-positive, tangle-like pathology in the soma of the DA neurons that was similar to NFTs seen in transgenic mice and AD brains. Flies expressing tau also exhibited motor and learning deficits that coincided with progressive neurodegeneration. Surprisingly, we observed that expression of htau^WT^ caused molecular pathogenesis in synapses, visualized by VMAT-pHluorin localization to nerve terminals that preceded identified pathological events such as tangle formation and overt cell loss. Our results suggest a potential molecular pathological mechanism through which tau may increase the risk of PD.

## Materials and methods

### Fly strains and genetics

Flies were cultured in standard corn meal–yeast–agar media at 25 °C, and 75 % relative humidity with a light/dark = 12/12 h cycle. *Ddc*-*Gal4* and *996TPH*-*GAL4* were kindly provided from Jay Hirsh (University of Virginia). *UAS*-*G*-*CaMP* was obtained from Richard Axel (Department of Biochemistry and Molecular Biophysics, Columbia University). *UAS*-*mCD8*-*GFP* and *GMR*-*GAL4* were obtained from Larry Zipursky (University of California, Los Angeles). Fly strains *TH*-*GAL4, DVGLUT*-*GAL4, GAD*-*GAL4, NPF*-*GAL4*, *RH1*-*GAL4, UAS*-*actinGFP* and *tubGAL80ts* were used in this study. *UAS*-*htau.1* (II), *UAS*-*htau.2* (III) and *UAS*-*VMAT*- *pHluorin* (III) were characterized in this study. *UAS*-*tau* ^*AP*^ *, UAS*-*tau* ^*E14*^ and *UAS*-*tau* ^*WT*^ were described previously [49, 50].

### Molecular cloning and transgenic flies

For generating human tau transgenes, the longest isoform of human tau cDNA, encoding amino acids 1–441, (Origene Technologies, Rockville, MD), was cloned into pUAST vector using *Eco*RI and *Not*I restriction sites. Site-directed mutagenesis was used to generate htau ^G272V^ and htau ^R406W^ with forward and reverse primer pairs of 5′-GAAGCACCAGCCGGGAGTC-3′ and 5′-CTTCCCGACTCCTG-3′ for G272V, and 5′-GACACGTCTCCATGGCATCTC-3′ and 5′-GAGATGCCATGGAGACGTGTC-3′ for R406W, respectively. The mutations of htau ^G272V^ and htau ^R406W^ were confirmed by DNA sequencing and subcloned into pUAST expression vector. Germ line transformation and standard fly-balancer crosses were performed. We obtained multiple *UAS*-*htau* ^*WT*^, *UAS*-*htau* ^*G272V*^ and *UAS*-*htau* ^*R406W*^ transgenic lines. Those with comparable expression level were selected for this study. PHluorin was inserted into the first luminal loop of VMAT and cloned into pUAST vector.

### Behavioral analysis

The startle-induced negative geotactic climbing assays were performed as previously described [2, 44]. For locomotion assay, 4-week-old flies (4 weeks after eclosion) were placed in chambers marked with grids and individual were recorded (videotaped) for periods of 10 min. Locomotion (locomotion index) was manually scored by counting the number of grids individuals crossed per minute. Olfactory associative learning was measured by training 3-week-old adult flies in a T-maze with the Pavlovian conditioning procedure as previously described [52]. Briefly, a group of 100 flies were first exposed to an odor (a conditioned stimulus, CS+) paired with 12 1.5 s pluses of 75 V DC electric shock (an unconditioned stimulus, US). For conditioning order stimulus, we used 3-octanol and 4-methyl-cyclohexanol. This was sequentially followed by presentation of a second odor (CS−) without reinforcement. Performance of conditioned avoidance responses was immediately measured with a choice between CS+ and CS− odors in a T-maze for 2 min after training. A performance index (PI) of aversive learning was calculated as (the number of flies in CS− arm) − (the number of flies in CS+ arm)/(the total number of flies) × 100.

### Confocal and electron microscopy

Fly brains and eyes were fixed, stained and mounted in Vectashield (Vector Laboratories, Burlingame, CA) as previously described [9]. Congo-philic stain was performed as previously describe [3]. Confocal microscopic observations of fly brains were performed using a Zeiss LSM 510 laser scanning confocal microscope (Carl-Zeiss, Germany). For characterization of different clusters of DA neurons, Z-stack confocal images that span 50 μm depth were collected from posterior to anterior direction to ensure all posterior clusters are covered. 3D images for each brain are used for examining neuron structure. For presenting purpose, Z-stack images are projected into a single 2D image.

For live brain imaging, fly brains were dissected in 1× PBS and G-CaMP was visualized immediately following brain dissection by confocal microscopy [54]. For VMAT-pHluorin experiments, released sites of VMAT-pHluorin of DA neurons were visualized as previous described [40]. Primary antibodies included mouse tau monoclonal antibodies AT8, AT100, and AT180 (Thermo Fisher Scientific, Waltham, MA), rabbit tau polyclonal antibody (Dako, Denmark), and mouse anti-TH (Immunostar, Hudson, WI). Secondary antibodies FITC and Cy3 conjugated anti-mouse or anti-rabbit (Jackson ImmunoResearch Laboratories, West Grove, PA) were used. NIH imageJ64 was used for quantification of G-CaMP and GFP using monochrome images, by measuring pixel intensity, as relative fluorescence with fluorescence-background calibration. For presenting G-CaMP activity, signal intensity was transformed to the pseudocolored thermal scale. Scanning electron microscopy was used to analyze fly eye morphology as previously described [10]. For pair-helical filament examination, transmission electron microscopy (TEM) was used as previously described [23], with the following modifications. Briefly, a resuspended sarkosyl insolubility pellet was placed on a carbon-coated copper grid (75 mesh) and stained with 1 % uranyl acetate. Samples were examined using HT7700 TEM (Hitachi).

### Western blots and sarkosyl insolubility assay

To detect the protein expression level, aged fly brains were homogenized in sample buffer using a glass micro-tissue grinder. To enrich tangles, sarkosyl-insoluble fractions of tau were purified from 40 homogenized fly heads as previously described [14] and used for western blot analysis and TEM observation. Standard western blotting procedures were used as previously described [8]. Primary antibodies included mouse cytochrome *c* (Gene Tex, Irvine, CA), and rabbit cleaved caspase 3 (Cell signaling technology, Danvers, MA). ECL reagents were used for antibody detection (Millipore, Billerica, MA) and imaging was performed using ImageQuant 350 (GE Healthcare).

## Results

### MAPT overexpression results in the progressive loss of DA neurons

Numerous mutations in MAPT have been linked to FTDP-17, including an increased ratio of the longest 4R Tau isoform [8]. In addition, several GWAS suggest tau variants as a risk factor for PD, and an increased incidence of PD is associated with elevated MAPT levels [16, 25, 32, 36, 38, 46, 55]. These observations suggest that misregulation of tau expression might contribute to the death of dopaminergic neurons. To investigate the effects of human tau overexpression in DA cells, we chose to test the effects of the largest htau^WT^ isoform (2N4R), because an increase of this largest isoform may predispose the susceptibility to PD or some FTDP-17.

**Fig. 1:**
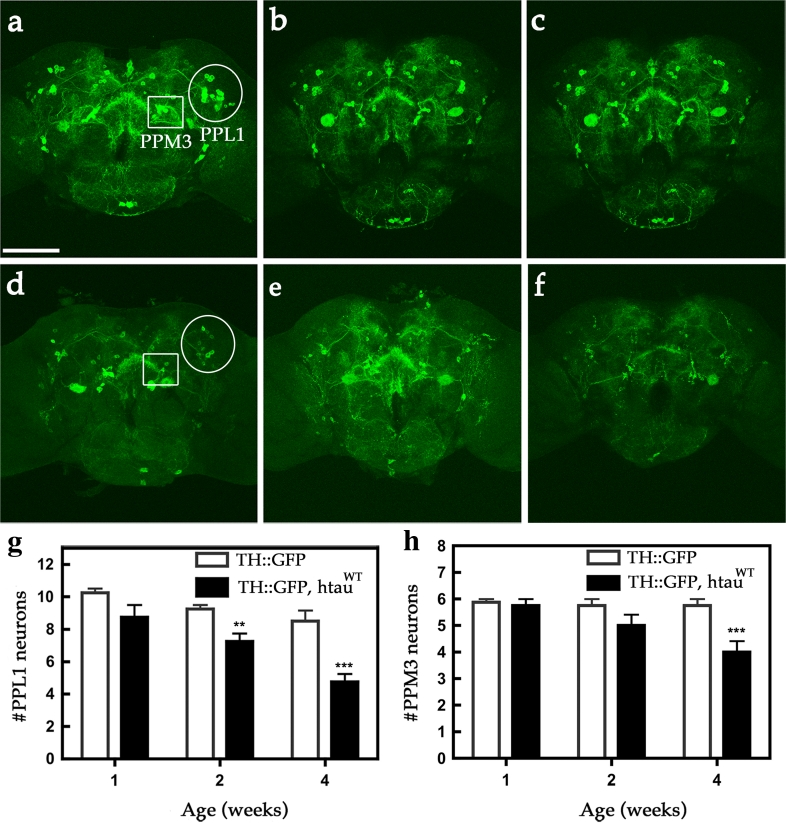
Expression of htau^WT^ induces age-dependent DA neuron demise. Representative confocal images show mCD8-GFP-marked DA neurons in age-matched control fly brains (**a**–**c** TH::mCD8-GFP) and htau^WT^-expressing brains (**d**–**f** TH::htau^WT^, mCD8-GFP) of one- (**a**, **d**), two- (**b**, **e**), and four- (**c**, **f**) week-old individuals. Two clusters of DA neurons, PPL1 (protocerebral posterior lateral 1, *circles*) and PPM3 (protocerebral posterior medial 3, *squares*), are indicated. *Scale bar* 100 μm. Cell counts of PPL1 (**g**) and PPM3 (**h**) DA neurons from age-matched control and htau^WT^ brains. Values shown represent Mean ± SEM (unpaired *t* test, **P < 0.001 at week 2 for **g**; ***P < 0.0001 at week 4 for **g** and **h**; *n* = 12)

Since the most affected neurons in PD are the DA neurons, we used the *TH*-*GAL4* driver to overexpress *UAS*-*htau* ^*WT*^ and co-express *UAS*-*mCD8*-*GFP*, to fluorescently label the plasma membrane of the cell body and neurite processes (TH::htau^WT^, mCD8-GFP). We then tested whether htau^WT^ expression resulted in degeneration of these DA neurons. In young adults (1-week-old), the number of GFP-marked DA neurons in htau^WT^ transgenic brains was comparable to control brains (TH::mCD8-GFP). At 2 weeks after eclosion, limited degeneration was observed in a small subset of PPL1 neurons expressing htau^WT^ compared to age-matched controls. Remarkably, at 4 weeks after eclosion, we observed a significant loss of DA neurons in transgenic flies relative to controls (Fig. 1). This loss occurred in two clusters of DA neurons: PPL1 and PPM3 (Fig. 1). Other FTDP-17 associated two tau variants of G272V and R406W, also showed similar effects (Fig. S1). To validate these htau^WT^-expressing cells may have undergone progressive degeneration, we characterized two components of the cell death signaling: cytochrome *c* and activated caspase 3-like caspase [53] for these htau^WT^-expressing cells and their GFP expressing controls in *Drosophila*. As expected, the expression of htau^WT^ produced an abnormal increase in the expression of cytochrome *c*, before a surge of caspase 3-like caspase activation in 3-week-old flies expressing htau^WT^, relative to controls, suggesting the activation of cell death signaling in htau^WT^-expressing brains in *Drosophila* (Fig. S2). These results indicate a progressive degeneration of DA neurons in the brains of *Drosophila* expressing htau^WT^. To further confirm the effect of htau^WT^, we employed a second GAL4 driver, *DDC*-*GAL4*, which targets htau^WT^ in both DA and serotonergic neurons. We confirmed that htau^WT^ does induce the age-dependent DA neuron degeneration (Fig. S3). Together, these results show that expression of htau^WT^ can induce progressive loss of DA neurons, manifesting the pathological DA degeneration in PD.

### MAPT overexpression produces age-dependent motor and learning deficits

**Fig. 2:**
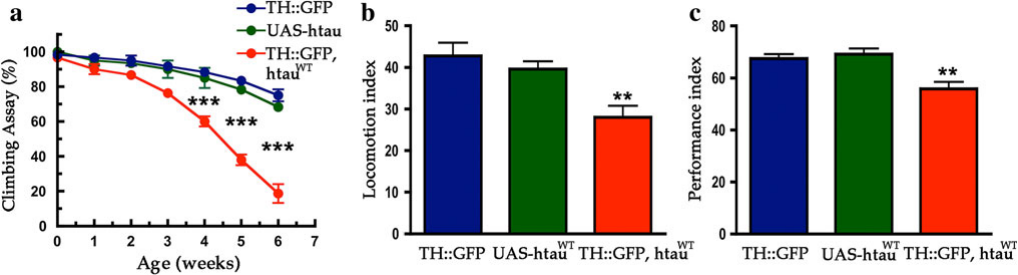
Expression of htau^WT^ in DA neurons causes motor and learning deficits. Behavioral analyses of driver control (TH::mCD8-GFP, *blue*), transgene control (*UAS*-*htau* ^*WT*^, *green*), and htau^WT^ expression (TH::htau^WT^, mCD8-GFP, *red*) flies. **a** Negative geotaxis assay was utilized for testing climbing activities of newly enclosed to 6 weeks age-matched adults. Accelerated decline of climbing activity was observed in flies expressing htau^WT^ in DA neurons (one-way ANOVA with Bonferroni’s multiple comparison test, ***P < 0.0001 compares each genotypes, *n* = 4). **b** Quantitative locomotion behaviors of 4-week-old flies show reduced activity in htau^WT^ expression group as compared to that in control groups (one-way ANOVA with Bonferroni’s multiple comparison test, **P < 0.001, *n* = 16). **c** Olfactory associative learning assay of 3-week-old flies revealed that flies in which DA neurons expressed htau^WT^ exhibited defects of olfactory aversive associative learning as compared to the control groups (one-way ANOVA with Bonferroni’s multiple comparison test, **P < 0.001, *n* = 4)

Similar to its role in mammals, the *Drosophila* nervous system utilizes many neurotransmitters to operate motor and cognitive behaviors. In the dopaminergic system, fruit fly use the same neurotransmitter as mammals to modulate a number of intricate neuronal circuits, ranging from those that control voluntary movement, mating, rewards, learning, and memory [41]. We tested whether expression of htau^WT^ would alter motor behaviors. Using a negative geotaxis assay, we observed that TH::htau^WT^ flies have a dramatically age-dependent climbing deficit; they showed significant loss of climbing ability compared to control flies at 4–6 weeks post-eclosion (Fig. 2a). In addition, we observed that flies expressing htau^WT^ showed reduced spontaneous locomotion; they appeared “sluggish” and exhibited an increase in the number of “pauses” between bouts of spontaneous locomotion and travelled shorter distances during each bout (Fig. 2b).

Next, we performed short-term olfactory association assays to test whether expression of htau in DA neurons affects learning. To avoid the potential confound caused by motor deficits, we tested 3-week-old cohorts, an age at which the motor behavior of htau^WT^-expressing flies is comparable to that of controls. As expected, we observed modest but significant learning deficit in htau^WT^-expressing flies, which scored 10 % lower than the age-matched control groups (Fig. 2c). This result is consistent with recent empirical findings indicating that dopaminergic neurons projecting to the mushroom body are essential mediators of olfactory associative learning and memory [1, 4, 7, 39, 42]. Together, these results show that flies expressing htau^WT^ in DA neurons manifest deficits in both motor and learning behaviors, which corroborate the observed age-dependent loss of DA neurons.

### Aminergic and NPF neurons are relatively more vulnerable to MAPT expression

**Fig. 3:**
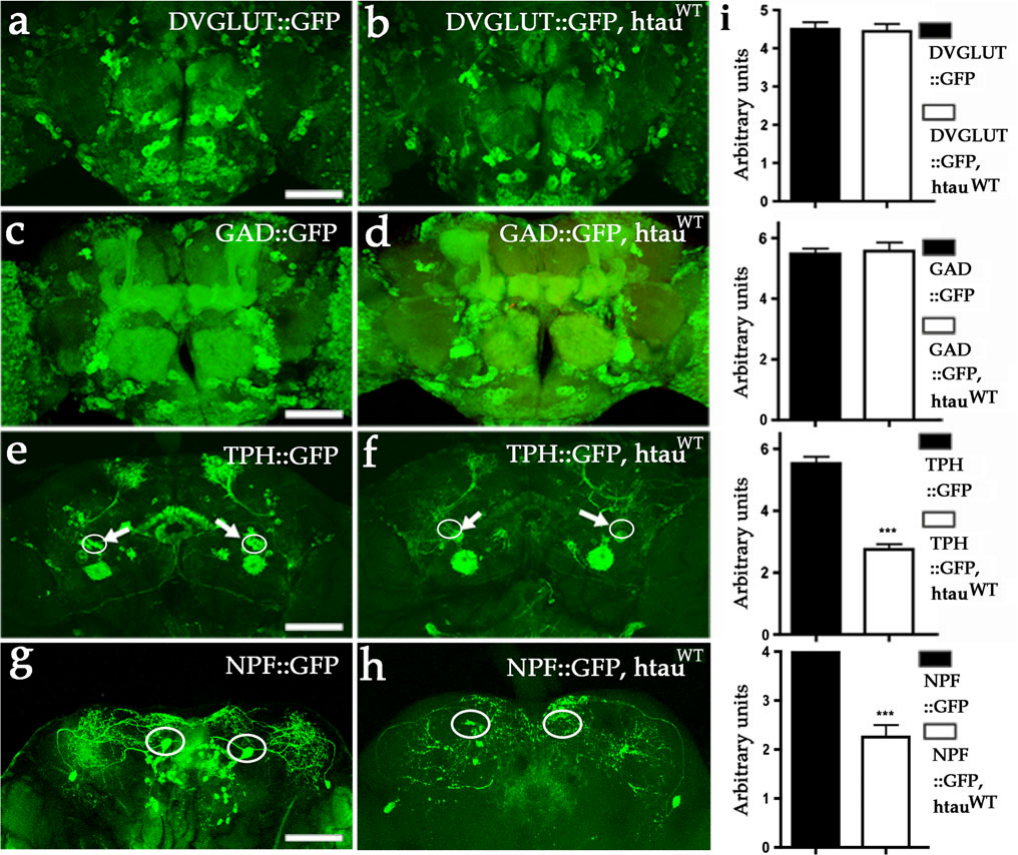
Expression of htau^WT^ fails to manifest neuronal loss in most glutamatergic and GABAergic neurons. **a**, **b** Representative confocal images show glutamatergic neurons of 4-week-old fly brains marked with mCD8-GFP. Several thousands of glutamatergic neurons with comparable GFP signals in both control (**a**, DVGLUT::mCD8-GFP) and htau^WT^ brains (**b**, DVGLUT::htau^WT^, mCD8-GFP). **c**, **d** Representative confocal images show mCD8-GFP-marked GABAergic neurons in 6-week-old fly brain. Comparable GFP signals can be detected in both control (**c** GAD::mCD8-GFP) and htau^WT^ brains (**d** GAD::htau^WT^, mCD8-GFP). **e** Representative confocal image shows a group of putative serotonergic neurons (TPH996::mCD8-GFP) projecting to central complex (*arrows*) at 4 weeks of age. **f** Representative confocal image shows the absence of GFP signals in neurons projecting to central complex in age-matched brain expressing htau^WT^ (TPH996::htau^WT^, mCD8-GFP). **g** Representative confocal image shows two pairs of putative NPF neurons (NPF::mCD8-GFP) projecting to parietal central brains in control (**f**). In contrast, the GFP signals in a pair of NPF neurons that are located in the anterior region are absent in htau^WT^-expressing brains (NPF::htau^WT^, mCD8-GFP) at 4 weeks of age. *Scale bar* 100 μm. **i** Quantitative analyses show no significant differences between control (*black bars*) and htau^WT^ (*white bars*) brains in DVGLUT, and GAD groups (unpaired *t* test, *P* = 0.54 for **a** and **b**; *P* = 0.60 for **c** and **d**, *n* = 4). In TPH and NPF groups, both the cell measurements (*circles*) between controls and htau^WT^ are different (unpaired *t* test, ***P < 0.0001 for both **e** and **f**, and for **g** and **h**, *n* = 4)

To determine how expression of htau^WT^ would affect neurons using other types of neurotransmitters, we used additional GAL4 drivers including *DVGLUT*-*GAL4* for glutamatergic neurons, *GAD*-*GAL4* for GABAergic neurons, *TPH*-*GAL4* for a subset of serotonergic neurons and *NPF*-*GAL4* for a subset of peptidergic F neurons. We did not observe loss of either GFP-marked glutamatergic or GABAergic cells (Fig. 3a–d, i); however, we observed that a subset of putative serotonergic neurons that project to the central complex and a subset of putative NPF neurons were lost (Fig. 3e–i). These results are consistent with previous studies that have shown human tau expression can cause degeneration of several neuronal populations in *Drosophila* [23, 33, 56]. Together, these results demonstrate that htau^WT^ can induce degeneration of DA neurons, serotonergic neurons, and NPF neurons, whereas most glutamatergic and GABAergic neurons are unaffected by htau^WT^ expression based on the fluorescent optical observation.

### Formation of abnormal oligomeric tau and tangle-like pathology in DA neurons

**Fig. 4:**
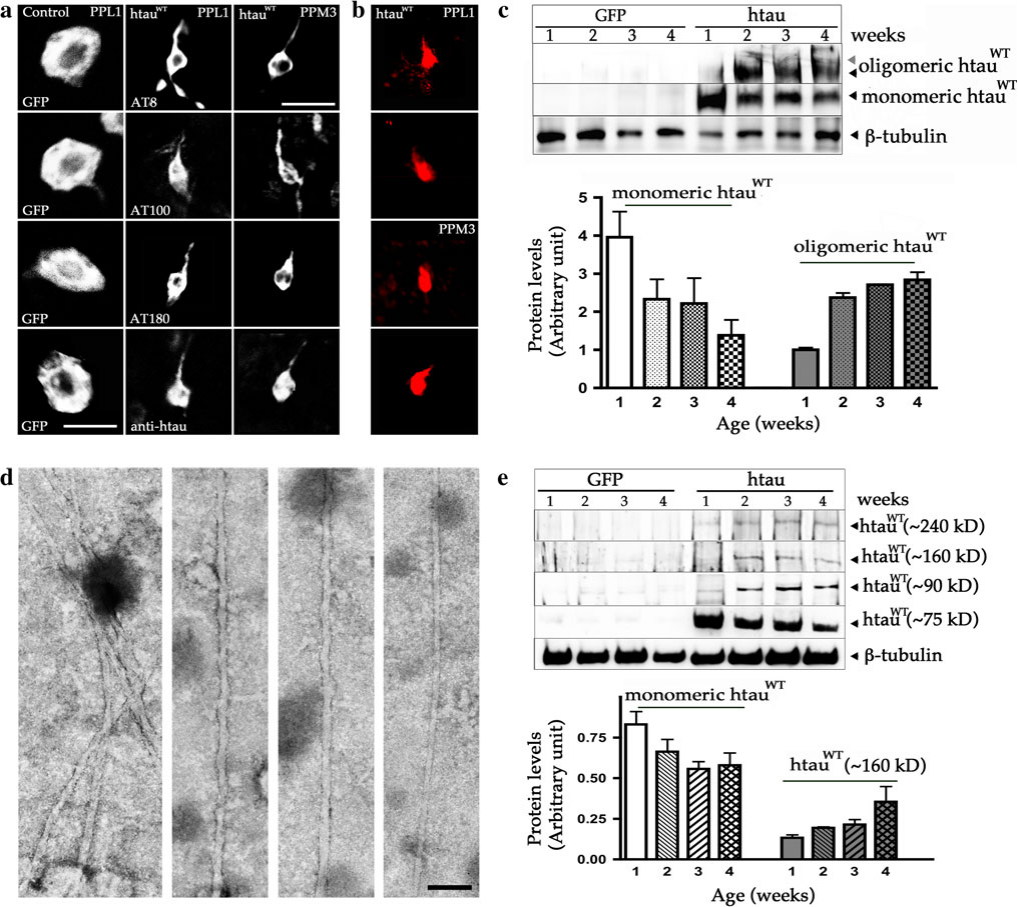
Expression of htau^WT^ produces AD-like abnormal tau phosphorylation and NFT-like pathology. **a** Single cell of PPL1 and PPM3 DA neurons from fly brains at 6 weeks of age. Compared to the age-matched control DA neurons from PPL1 cluster labeled with GFP (*left column*), immunostaining of three antibodies that recognize AD-like hyperphosphorylated tau (AT8 *top row*, AT100 *top second row*, AT180 *bottom second row*), and a polyclonal tau antibody (*bottom row*) reveal tangle-like pathology in the degenerating DA neurons from PPL1 (*second column*) and PPM3 (*third column*) clusters. **b** The degenerating DA neurons from both PPL1 and PPM3 clusters are stained with Congo red. The time course of pathological tangle-like structure formation in htau^WT^ brains during the aging process is presented in Fig. S3. **c** Representative immunoblot of polyclonal anti-htau shows that a substantial amount of htau^WT^ proteins are converted from monomer tau (75 kDa) to the oligomeric Tau species (>400 kDa). Anti-β-tubulin serves as a loading control. Protein expression levels from four independent immunoblots with monomeric and oligomeric htau protein levels calibrated against the loading control are shown as relative expression. Values shown represent mean ± SEM. **d** Electron micrographs of abnormal filaments extracted from tangle-rich preparation (sarkosyl-insoluble pellets) of fly brains expressing htau^WT^. Images show several PHF-like filaments are bundled (*left panel*), a single PHF (*middle panels*), and a straight filament (*right panel*). *Scale bar* 100 nm. **d** Representative immunoblot from sarkosyl-insoluble pellets reveals monomer tau ***(75 kD) and the htau-containing species with molecular weights range between 75 and 400 kD (~90, ~160 and ~240 kD). Anti-β-tubulin also serves as a loading control. **e** Monomeric and polymeric htau protein levels are quantified by analyzing three independent immunoblots and shown as relative expression. Values represent mean ± SEM

Hyperphosphorylated tau has been suggested to accumulate as homogeneous or heterogeneous oligomers and subsequently form NFT [5]. To determine whether similar pathogenesis occurs in DA neurons that express htau^WT^, we used three common phosphoepitope antibodies (AT8, AT100, and AT180) and a polyclonal anti-tau antibody that have been widely utilized to characterize AD-like abnormal phosphorylation of tau in the postmortem brains of AD patients and animal models of tauopathies. Immunohistochemical analysis of TH::htau^WT^ brains at different ages revealed that the morphology of DA neurons appeared normal in the first week, with no detectable morphological changes in the soma and neurites, suggesting that the neuronal architecture was normal in young adults (Fig. S4). At approximately 2 weeks post-eclosion, the staining pattern of the soma in some DA neurons has changed relative to the wild-type morphology, indicating that the cytoskeletal networks had been perturbed or disintegrated by this time (Fig. S4). Strikingly, after 3 weeks, the phosphoepitope antibodies and a polyclonal anti-tau antibody began to reveal abnormal tangle-like morphology, resembling NFTs at the microscopic level in most DA neurons including those in PPL1 and PPM3. This result suggests that the appearance of abnormal NFTs in DA neurons is an age-dependent process (Fig. S4). To explore whether htau expression might also induce tangle pathology in general, we investigated other types of neurons expressing htau^WT^. Similar morphological change was also detected in a small subset of 5HT neurons, but no obvious alteration was detected in glutamatergic, GABAergic and NPF neurons in 6-week-old flies (Fig. S4). DA neurons that express htau^WT^ exhibited largely pruned neurite branches at the distal end in 4-week-old individuals (Fig. S4). The few cells that survived until 6 weeks experienced soma shrinkage, which was dramatically different from age-matched, GFP-labeled DA neurons from control brains (Fig. 4a, S4). Moreover, we confirmed that at 6 weeks, these abnormal DA neurons expressing htau^WT^ were stained by Congo red, a synthetic dye which recognized the abnormal β-sheet conformation of htau aggregations (Fig. 4b).

To determine whether these morphological changes were associated with structural changes in htau^WT^, we performed additional biochemical analyses on whole brain extracts from aged flies expressing TH::htau^WT^. We observed a gradual reduction of monomeric tau, and an increase in high-molecular-weight species, of human tau protein with increasing age (Fig. 4c). The appearance of the putative tau oligomers (>400 kDa) seemed to coincide with the onset of morphological change in DA neurons, which is in agreement with the hypothesis that tau oligomers may be toxic.

In order to more rigorously evaluate htau aggregates in DA neurons, we performed ultrastructure examination on sarkosyl-insoluble fraction extracted from htau transgenic brains. TEM revealed abundant filamentous aggregates formed in the extracts from fly brains of 4-week-old *Drosophila* expressing htau^WT^ (Fig. 4d), but not in identically prepared sarkosyl-insoluble fraction from age-matched nontransgenic control brains. These negatively stained filaments were either helical (paired helical filaments, PHF) or straight (paired straight filaments, SF), with diameters of approximately 15–40 nm and the axial distance between consecutive wider portions of approximately 80–100 nm (Fig. 4d), resembling those PHFs found in AD [12]. Therefore, we suggest that the observed htau aggregates with an NFT-like pathology may be composed by PHF or SF.

To determine whether htau^WT^ insolubility coincided with the appearance of the tangle-like morphology in DA neurons, we performed sarkosyl insolubility assays from brains at different ages. Western blotting of anti-htau revealed a modest reduction of insoluble tau monomer, and an increase with age, of several insoluble high-molecular-weight species between 75 and 400 kDa, including ~90, ~160, and ~240 kDa tau-positive bands (Fig. 4e). The formation of these insoluble high-molecular-weight aggregations apparently preceded the formation of tangles, which is consistent with the hypothesis that insoluble tau-containing aggregations may be responsible for the NFT-like pathology.

### Effects of tau phosphorylation on neuron degeneration

Previous studies have suggested that tau phosphorylation is associated with AD and other tauopathies, although the role of phosphorylated state in disease pathogenesis remains controversial. To test whether the htau^WT^-induced DA neuron degeneration in *Drosophila* is related to its phosphorylation state, we expressed htau^WT^ and phosphorylation site variants in DA neurons and compared their potential toxicity in aged fly brains. We focused on SP/TP sites, for which several proline-directed kinases such as glycogen synthase kinase 3 beta (GSK-3β) have been shown to modulate tau-mediated toxicity [23], and compared the effects of htau^WT^, htau^AP^, and htau^E14^. The AP mutation blocked proline-directed tau phosphorylation and the E14 mutation mimics phosphorylation of the endogenous 14 serine/threonine amino acids in htau. To confirm that htau^WT^ can undergo phosphorylation in *Drosophila* DA cells, we performed western blots and observed a shift in the molecular weight of htau^WT^, from 75 to 60 kDa, in response to treatment with alkaline phosphatase (Fig. S5).

**Fig. 5:**
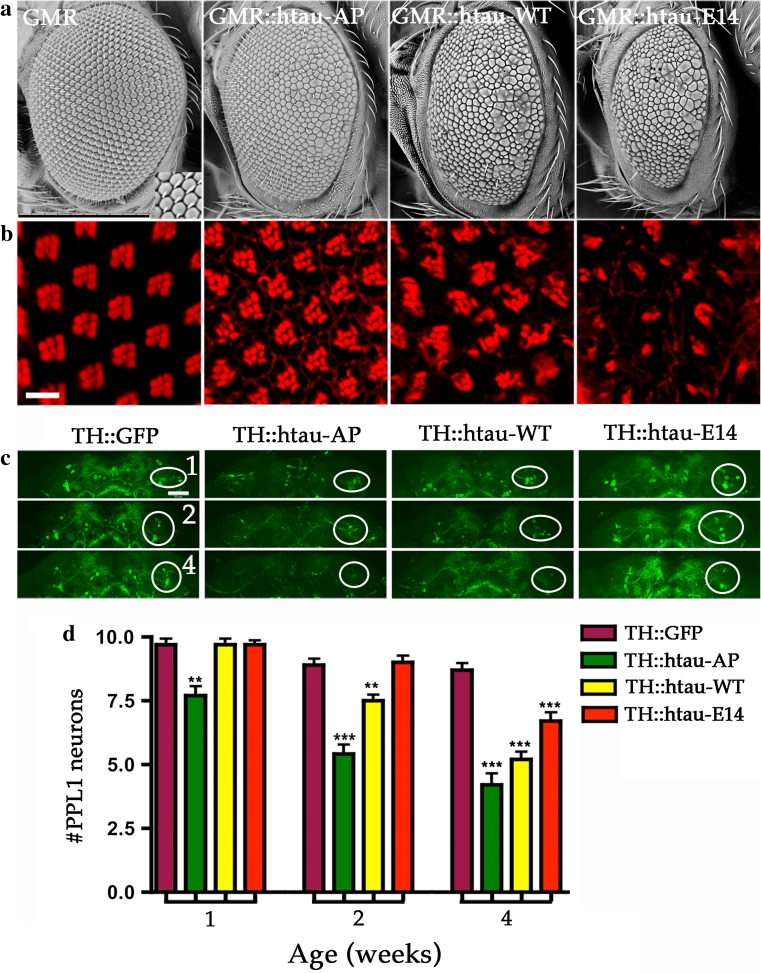
Different phosphorylation site mutations of tau exert contrary effects on DA and histaminergic neurons. Representative SEM (**a**) and confocal (**b**) images of fly compound eyes. Control (*GMR*-*GAL4*) fly eyes show crystalline array of ~800 hexagonal-shaped ommatidia form the smooth external surface. Phalloidin staining reveals the underlying organization of each ommatidium with actin-rich rhabdomeres from seven photoreceptors being arranged in a trapezoid format. Expression of three human tau transgenic alleles, htau^AP14^ (GMR::htau-AP), htau^WT^ (GMR::htau-WT), and htau^E14^ (GMR::htau-E14), produce different degrees of eye phenotypes and the severity ranking of eye defect is htau^E14^ > htau^WT^ > htau^AP14^ that is based on external eye size and roughness and the internal organization of photoreceptor cells (**a**, **b**). *Scale bars* 10 μm. **c** Representative confocal images of control DA neurons marked with mCD8-GFP (TH::GFP), or co-expressing with either of the three human tau alleles (htau^AP14^, TH::htau-AP; htau^WT,^ TH::htau-WT; and htau^E14^, TH::htau-E14) from 1-, 2-, and 4-week-old brains. *Circles* indicate the PPL1 cluster. **d** Quantitative analysis presents DA neuron numbers in PPL1 cluster at different ages from control and three tau alleles. TH::GFP (*purple*), TH::htau-AP (*green*), TH::htau-WT (*yellow*) and TH::htau-E14 (*red*). Values shown represent mean ± SEM (unpaired *t* test compares individual tau allele to control in the age-indicated groups; **P < 0.001; ***P < 0.0001, *n* = 12)

An earlier study showed that the htau^AP^ mutation is less toxic than htau^WT^ when expressed in *Drosophila* photoreceptors, but that the htau^E14^ mutation enhances neurotoxicity in the retina [49, 50]. Our results, using *GMR*-*GAL4* driver to express these htau constructs in the retina, confirmed these observations (Fig. 5a, b). Surprisingly, the htau constructs appeared to have inverse effects when expressed in DA cells using TH-GAL4. TH::htau^AP^ brains showed an increase in DA cell death, compared to flies expressing htau^WT^, and htau^E14^ resulted in less neurodegeneration than tau^WT^ (Fig. 5c, d). Western blots validated that htau^WT^, htau^AP^, and htau^E14^ were expressed at similar levels, indicating that the phenotype is specific for the mutation in tau rather than expression of the transgenes (Fig. S5).

It is possible that the severe neuronal phenotype induced by htau^AP^ is a developmental, but not a degenerative event. To circumvent potential developmental effects of htau^AP^ on DA neurons, we used a conditional expression system by incorporating temperature-sensitive GAL80^ts^ [27] in our model to predominantly, if not exclusively, expressing htau^AP^ in adult brains. By shifting temperature from 25 to 29 °C for newly enclosed adult flies. We induced transgene expressing, and observed that adult onset of htau^AP^ expression (*GAL80* ^*ts*^/*UAS*-*htau* ^*AP*^; *TH*-*GAL4, UAS*-*CD8GFP/*+) induced severe neurodegeneration, similar to that of the continuously expression of htau^AP^ (TH::htau^AP^, GFP), while the control flies (TH::GFP) exhibited normal DA neurons (Fig. S6). Therefore, these data suggest the effect of htau^AP^ on DA neuron was an age-dependent degeneration, but not a developmental defect.

### Loss of vesicular dopamine release preceded tauopathy and neurodegeneration

Previously, we have shown that vesicular monoamine transporter (VMAT) reduced environmental toxins and mutant human parkin-induced toxicity [26, 44]. We reasoned that the loss of DA neurons resulting from the expression of human tau may be associated with a change in the DA storage and release machinery. To investigate this, we used the ecliptic VMAT-pHluorin, with a pH-sensitive GFP conjugated in the first luminal loop of the 12 trans-membrane domains of VMAT to visualize the release of VMAT-pHluorin containing synaptic vesicle released to nerve terminals [34].

**Fig. 6:**
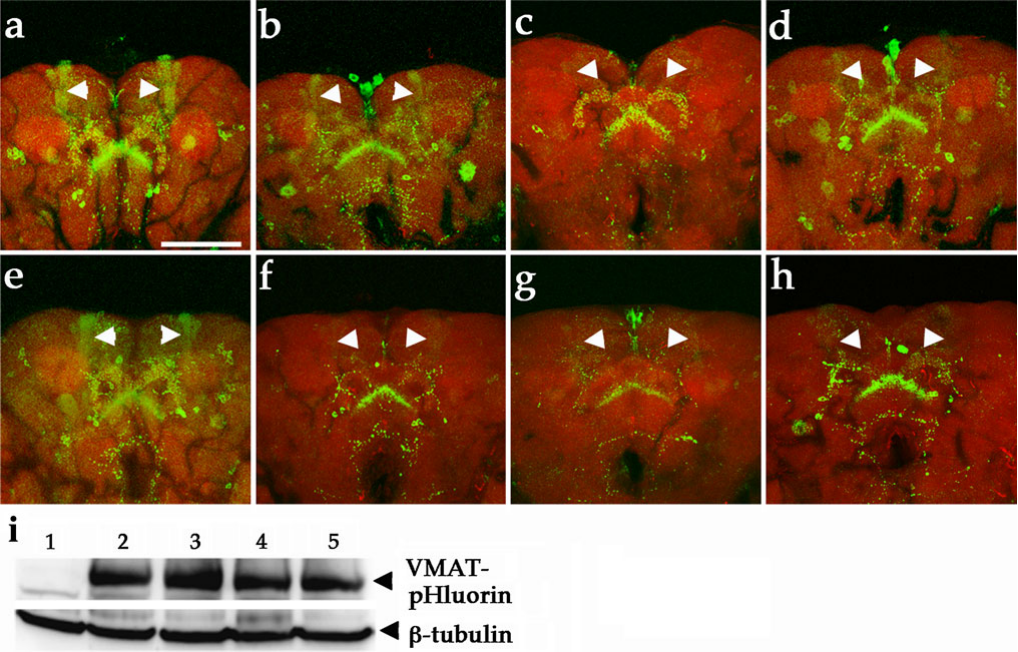
DA neurons expressing htau^WT^ cause early impairment of vesicular dopamine release to nerve terminals as visualized by VMAT-pHluorin. **a**–**f** Confocal images of DA neurons from brains of 1-day-old (**a**–**d**) and 3-week-old (**e**–**h**) flies show VMAT-pHluorin reporter (*green*). **a** DA neurons expressing VMAT-pHluorin (TH::VMAT-pHluorin) reveal localization of VMAT-pHluorin to presynaptic terminals in brain regions, and **b** age-matched DA neurons co-expressed VMAT-pHluorin and htau^WT^ (TH::VMAT-pHluorin, UAS-htau^WT^) show decreased GFP signals in mushroom bodies (*arrowheads*). **e** A representative confocal image shows the VMAT-pHluorin signals to mushroom bodies and other brain structure remains prominent (*green*) in 3-week-old control brain, while the GFP signals localized to mushroom bodies is diminished in DA neurons expressing htau^WT^ (**f**, *arrowheads*). **c**, **g** Expression of htau^AP^ evokes severe loss of pHluorin signaling compared to age-matched tau^WT^ (**b**, **f**) and Tau^E14^ (**d**, **h**). Rhodamine-phalloidin (*red*) marks the brain structure. *Scale bar* 100 μm. **i** Representative western blot shows the expression levels of VMAT-pHlurion of 1-week-old fly brains in control and experiment groups. *1* *TH*-*GAL4*, *2* TH::VMAT-pHluorin, *3* TH::VMAT-pHluorin, htau^WT^, *4* TH::VMAT-pHluorin, htau^AP^, *5* TH::VMAT-pHluorin, htau^E14^. Anti-β-tubulin served as a loading control

In wild-type fly brain, VMAT-pHluorin visualizes the synaptic vesicle-releasing regions of DA neurons in the fly brain. At one day after eclosion, VMAT was observed in many brain regions, including mushroom bodies, the fan-shaped body, and the protocerebral bridge, in wild-type flies (Fig. 6a) [31]. The single neuron of PPL1 and PPM3 data has been deposited in the database of *Drosophila* neurons (http://www.flycircuit.tw). In contrast, the localization of VMAT to the mushroom body and protocerebral bridge was reduced in age-matched flies expressing htau^WT^ (Fig. 6b). This decrease was most pronounced in 3-week-old fly brains compared to the wild-type fly brain (Fig. 6e, f). Remarkably, the VMAT-pHluorin signals in the mushroom body and protocerebral bridge were selectively absent from the htau^AP^ brains at one-day post-eclosion, but remained prominent in controls (Fig. 6a, c). Interestingly, some neurons that project to the fan-shaped body appeared to be unaffected, as the VMAT-pHluorin signal appeared normal in both control and three htau (htau^WT^, htau^AP^ and htau^E14^) expressing brains. Given that some *TH*-*GAL4*-marked neurons are not dopaminergic, we suspect that the unaffected VMAT-pHluorin labeling of the fan-shaped body might be a result of expression in those cells. To further investigate whether declines in VMAT-pHluorin localization to nerve terminals were indeed related to human tau-induced toxicity, DA neurons expressing of htau^WT^, htau^AP^, or htau^E14^, were compared in different ages (Fig. 6a–h). Surprisingly, the age-matched flies expressing htau^AP^ had earlier and more profound loss of VMAT-pHluorin signals (Fig. 6c) than flies expressing htau^WT^ or htau^E14^ (Fig. 6b, d). Variation in the GFP signals in brains co-expressing VMAT-pHluorin and htau^WT^ or htau^AP^ were not a result of variation in levels of VMAT-pHluorin expression, since levels of VMAT-pHluorin were similar in both experimental and control groups (Fig. 6i).

**Fig. 7:**
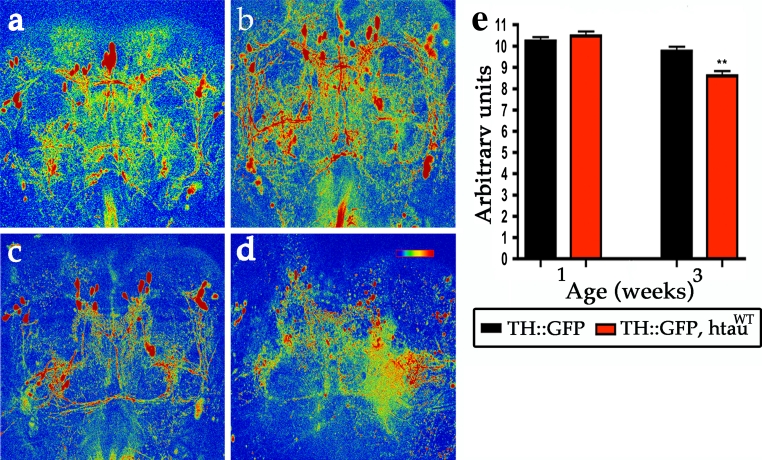
Expression of htau^WT^ causes age-dependent loss of G-CaMP activities. G-CaMP signal reveals Ca^2+^ activity in control DA neurons (TH::G-CaMP) from 1-week-old (**a**) and 3-week-old (**c**) flies. Expression of htau^WT^ shows comparable G-CaMP signals compared to control group at 1 week (**b**), but the signals are reduced 10 % at 3 weeks (**d**). The G-CaMP activities are represented by thermal color from the higher (*red*) to lower intensity (*blue*). *Scale bar* 100 μm. **e** Quantitative analysis shows G-CaMP activities in control brains (TH::G-CaMP, *black bars*) and in htau^WT^ brains (TH::G-CaMP, htau^WT^, *red bars*) at indicated ages. Values shown represent Mean ± SEM (unpaired *t* test; **P < 0.001)

It is possible that htau could cause a general dysfunction of DA neurons resulting in multiple deficits in the DA release machinery. To determine whether expression of htau^WT^ also disrupts calcium influx, we used a calcium-sensitive fluorescent protein, G-CaMP, to monitor changes in calcium. By visualizing 1-week-old live brains, we found that expression of htau^WT^ did not alter the G-CaMP activity, compared to controls (Fig. 7a, b, e), suggesting that htau^WT^ expression did not disrupt the general function of DA neurons during neuronal activation. However, at 3 weeks, we found that DA neurons expressing htau^WT^ showed a minor but significant reduction of the G-CaMP signal compared to controls (Fig. 7c–e), consistent with overt DA neuronal degeneration around this age (Fig 7c–e). In conclusion, these results suggest htau^WT^ inhibits the VMAT-containing synaptic vesicle release to neuronal processes, before overt neuronal degeneration.

## Discussion

In the present study, we used *Drosophila* DA neurons as a model to examine the pathogenic mechanism of PD-linked tauopathies. Expression of htau^WT^ in these neurons resulted in age-dependent, progressive neurodegeneration and the deposition of abnormal polymeric tau protein aggregates. Systematic characterizations of functional, pathological, and behavioral readouts from these htau^WT^ flies at different ages demonstrate a plausible pathogenic link between tau and vesicular DA release, as demonstrated by a fluorescent VMAT-pHluorin marker in the synaptic vesicle-releasing sites of DA neurons. This defect represents the earliest pathological manifestation that precedes the robust pathophysiological phenotypes and suggests that a functional inhibition of DA neurotransmission is a prelude of the hallmark lesions in our PD model.

The molecular components of DA signaling and homeostasis in *Drosophila* are similar to those in humans; both human and fly DA neurons express two types of transporters: (1) a vesicular monoamine transporter for transport of DA from the cytosol to the lumen of secretory vesicles [29]; (2) a plasma membrane DA transporter (DAT) for reuptake of exocytosed DA [18]. Therefore, despite the anatomical differences, *Drosophila* models of PD, as well as other human neurological disorders, can help contribute to our understanding of the pathogenic mechanism underlying these diseases [6]. This tau-PD *Drosophila* model is the first animal model to demonstrate that tau expression can cause degeneration of DA neurons, a hallmark of Parkinson’s disease.

Most motor symptoms and mild cognitive impairment of PD result primarily from progressive degeneration of DA neurons in the substantia nigra, but the mechanism through which pathogenic factors target DA neurons is not completely understood. While several toxic compounds are known to produce PD symptoms in animal models, only MPTP (1-methyl-4-phenyl-1,2,3,6-tetrahydropyridine) has been firmly established as a cause of selective DA neuron toxicity. The issue of neuronal vulnerability, in terms of transporter or transmission mechanisms among individuals with monogenic PD risk factors, has rarely been addressed directly. One exception is a study that showed that the expression of human α-synuclein in mice inhibited DA transmission by reducing the recycling of synaptic vesicles [37]. In addition, an association between DA metabolism and the death of DA neurons in PD has been postulated, and this association has been demonstrated in a α-synuclein PD model. Oxidative metabolites of DA may conjugate with α-synuclein to form an adduct of DA–α-synuclein, which may stabilize the toxic form of α-synuclein through covalent bound to DA quinone [11], while also promoting selective neurotoxicity [58]. Tau protein is ubiquitous in the brain, and has been shown to affect different types of neurons in various cell and animal models. A previous study showed that DA quinones, toxic metabolites that lead to PD, could promote the assembly of tau into fibrillar polymeric tau in vitro [15] which suggests a potential interplay between DA and tau in vivo and may provide a basis for the vulnerability of DA neurons.

One of the common themes of tau pathology is abnormal hyperphosphorylation of tau, originally identified in AD patients. However, the role of tau hyperphosphorylation in the pathophysiology of disease remains debated. In *Drosophila* photoreceptors, tau hyperphosphorylation is correlated with phenotypic severity [49], similar to the results observed in this study. However, we also determined that in DA neurons, the hypophosphorylated AP mutation increases toxicity while the hyperphosphorylated E14 mutation reduces toxicity. These data indicate that tau pathogenesis is likely influenced by multiple pathological mechanisms, because its phosphorylation mutations in DA and histaminergic neurons produce contrasting results. The hyperphosphorylation E14 mutation has a reduced binding affinity for microtubules, whereas the hypophosphorylation AP mutation has an increased affinity for microtubules. Thus, we suspect that hypophosphorylation AP tau mutation may profoundly block axonal transport and consequently inhibit synaptic vesicle transport. These notions raise the possibility that microtubule bound tau may partially contribute to tau pathogenesis in DA neurons due to its association with severity of phenotype in DA neurons; they are consistent with the concept that tau can compete with microtubule motor proteins and lead to the detachment of motor proteins (kinesin and dynein), which may lead to abnormal axonal trafficking [51]. However, since this tau^AP^ is a tool to study the relationship of dephosphorylation state and disease severity in *Drosophila*, it may have little relevance to pathophysiology of tau in PD because tau is likely hyperphosphorylated in PD. Although the precise pathogenic mechanism of tau on DA neuron degeneration remains unclear, tau may interfere with the transport of synaptic vesicles and organelles such as mitochondria along microtubules [30]. Interestingly, when tau was overexpressed in photoreceptors at late pupal stage, we observed that transport of rhodopsin to the photosensitive membrane, rhabdomere, was inhibited (Fig. S7). Here, we show that the localization of VMAT to nerve terminals is also impeded. VMATs have been shown to exert neuroprotective effects in DA neurons in both flies [26] and mammals presumably via sequestration of cytosolic DA [19, 35]. We postulate that decreased localization of VMAT to the nerve terminal caused by tau could contribute to the pathogenesis of PD.

We show that the expression of human tau evokes aberrant polymeric tau formation, during which PHF-like aggregates accumulate and NFT-like tangles develop in DA neurons; this occurs in an age-dependent manner. This is the first longitudinal study to directly examine tau tangle formation and other pathogenesis in DA neurons. In contrast to previous studies which show that tauopathy can evoke neuronal degeneration but cannot form neurofibrillary tangles in other types of neurons in *Drosophila* and *Caenorhabditis* [24, 56]; our results suggest that human tau can produce tangle pathology in DA neurons, similar to the tau neurofibrillary tangles seen in PD patients. We suspect that the difference of tau manifestations is likely due to dopamine modifications and perhaps different amount of tau proteins in each neuron and believe that our tau-PD *Drosophila* model offers a robust animal model that can facilitate to investigate modification of tau pathogenesis.

Our neuropathological observations reveal at least two distinct molecular mechanisms of tau pathogenesis in DA neurons for PD. We show that failure of VMAT-containing synaptic vesicle release to nerve terminals is an early pathogenic manifestation, and the formation of neurofibrillary tangle-like pathology in the soma of DA neurons is a late-stage of tauopathy, providing pathological evidence for why *MAPT* may be a strong risk factor for idiopathic PD. The correction or prevention of the deficit in vesicular DA storage may be appropriate target for early therapeutic intervention.

## Electronic supplementary material

Below is the link to the electronic supplementary material.

Supplementary material 1 Expression of htau^G272V^ and htau^R406W^ cause DA neuron degeneration similar to htau^WT^. Related to Fig 1. (a) A depicted human tau isoform with 441 amino acids. The N domains are shown in white boxes and the C-terminal microtubule binding repeats are shown in black boxes with labeled as R1-R4. Two FTDP-17 associated mutations, G272V and R406W, are indicated. (b-e) Representative confocal images show mCD8-GFP-marked DA neurons in four-week-old control fly brain (b, TH::mCD8-GFP), and age-matched brains from flies expressing htau^WT^ (c, TH:: htau^WT^, mCD8-GFP), htau^G272V^ (d, TH:: htau^G272V^, mCD8-GFP), and htau^R406W^ (e, TH:: htau^R406W^, mCD8-GFP). Two clusters of DA neurons, PPL1 (circles) and PPM3 (squares), are indicated. (f) Representative western blot shows protein levels of htau^WT^, htau^G272V^, and htau^R406W^ that expressing in DA neurons. No human tau proteins can be detected in TH::mCD8-GFP and UAS-htau^WT^ controls. β-Tubulin serves as a loading control. (g) Quantification of four independent western blots. Values shown represent Mean ± SEM; one-way ANOVA, P = 0.6185; ns, not significant. (TIFF 3756 kb)

Supplementary material 2 Expression of htau^WT^ activates cell death signaling. (a) Representative immunoblot shows increased cytochrome c and activated caspase 3-like caspase in brains with DA neurons that expressing htau^WT^ (TH::htau^WT^) compared to age-matched control (TH::GFP). The decrease of caspase 3-like signal at the fourth week is likely influenced by DA neuron loss by this age. Anti-ß-tubulin serves as a loading control. Quantification from four independent immunoblots shows expression levels of cytochrome c (b) and activated caspase 3 (c) represented as relative expression. Values shown represent Mean ± SEM. (TIFF 923 kb)

Supplementary material 3 Expression of htau^WT^ in DA and 5HT neurons also induced DA neuron degeneration. Related to Fig 1. (a) Representative confocal images of control fly brains (left panels, DDC::mCD8-GFP) and brains expressing htau^WT^ (right panels, DDC::htau^WT^, mCD8-GFP) at different ages (weeks numbered in the upper right corner of the left panels) stained with anti-tyrosine hydroxylase (anti-TH, red). PPM2 clusters of DA neurons (circles) are marked with both anti-TH and GFP (yellow). Scale bar, 50 µm. (b) Immunoblotting of a polyclonal Tau antibody (against the C-terminal amino acids 243-441) detects a 75 kDa band in DDC:: htau^WT^, but not in DDC-GAL4 or UAS-htau^WT^ controls. Anti-tubulin served as a loading control. (c) Quantitative analysis shows the number of DA neurons in PPM2 clusters in htau^WT^ (pink) and control (green) at indicated ages. Values shown represent Mean ± SEM (unpaired t-test, *P < 0.01; ** P < 0.001; ***P < 0.0001, n = 10). (TIFF 2561 kb)

Supplementary material 4 The time course of developing tangle-like pathology in the degenerating DA neurons and the effect of htau^WT^ on other types of neurons. Related to Figure 4. (a) Confocal images focusing at a single DA neuron from control (TH::mCD8-GFP) and htau^WT^ expressing (TH-GAL4:: UAS-htau^WT^, mCD8-GFP) brains at indicated ages (in week indicated at the lower left corner of row). GFP-marked DA neuron in control brains shows comparable soma size at different ages (a), while DA neuron expressing htau^WT^ reveals progressive reduction of soma size as well as the formation of tangle-like pathology extended from the soma begin at approximate 3 weeks of age, featured by AD-like hyperphosphorylated tau antibodies AT8 and AT180 staining and a polyclonal Tau antibody (a). (b) Confocal images of a single DA neuron marked by actin-GFP reveal edges of the soma are associated with actin rich puncta in the control brain (TH::actin-GFP), but are absent of these actin rich puncta on the surface of the soma in the htauWT expressing brain. Confocal images of AT8 immunostaining show the fragmented axonal branches (arrows) at 4 weeks (c) and 6 weeks (d) htau^WT^ expressing DA neurons. (e) Other types of neurons expressing htau^WT^ show comparable soma size and shape (right panels) as compared to normal glutamatergic (top row), GABAergic (second row from top) and NPF (bottom row) neurons (left panels). In contrast, 5HT neurons expressing htau^WT^ show reduction in soma size and morphological change, similar to that of DA neurons in 6-week-old flies. Scale bar, 10 µm. (TIFF 3941 kb)

Supplementary material 5 Analysis of phosphorylation states of htau^WT^ proteins and protein levels of three human tau alleles: htau^AP^, htau^WT^, and htau^E14^ in DA neurons. (a) Representative western blot shows expression of htau^WT^ protein in DA neurons were phosphorylated (-) and the migration in SDS-PAGE is slower than those htau^WT^ proteins treated with alkaline phosphatase (+). No human tau proteins can be detected in control TH::GFP. β-Tubulin serves as a loading control. (b) Representative western blot shows protein levels of three htau alleles in DA neurons: htau^WT^ (TH::mCD8-GFP, htau^WT^), htau^AP^ (TH::mCD8-GFP, htau^AP^), and htau^E14^ (TH::mCD8-GFP, htau^E14^). β-Tubulin serves as a loading control. (c) Quantification of three independent western blots. Values shown represent Mean ± SEM; one-way ANOVA, P = 0.925; n = 3). (TIFF 474 kb)

Supplementary material 6 Adult onset of htau^AP^ expression also produces severe age-dependent neurodegeneration. Related to Figure 5. (a) Representative western blot shows conditional expression of htau^AP^. At room temperature (25°C), tub-GAL80ts represses transgene expression, thus htau^AP^ is silenced (TH::htau^AP^,mCD8-GFP/tubGAL80ts at 25°C); whereas at 29°C, tub-GAL80ts fails to repress transgene expression, thus the expression of htau^AP^ transgene is detected (TH::htau^AP^, mCD8-GFP/tubGAL80ts at 29°C). No htau^AP^ is detected in control (TH::mCD8-GFP). (b) Representative confocal images of PPL1 groups of DA neurons marked with mCD8-GFP (TH::GFP) in the control, conditional expression of htau^AP^ in adult flies (TH::htau^AP^, mCD8-GFP/tubGAL80ts), or developmental expression of htau^AP^ (TH::htau^AP^, mCD8-GFP) at indicated ages. Circles indicate the PPL1 clusters. (c) Quantitative analysis presents DA neuron numbers in PPL1 cluster at different ages (weeks) from control and htau^AP^ with indicted expression regimes. TH:: mCD8-GFP (blue); TH::htau^AP^, mCD8-GFP/tubGAL80ts (yellow); TH::htau^AP^, mCD8-GFP (pink). Values shown represent Mean ± SEM (unpaired t test compares individual tau allele to the control in the age-matched groups; *P < 0.01; ***P < 0.0001, n = 12). (TIFF 1482 kb)

Supplementary material 7 Expression of htau^WT^ causes the mislocalization of rhodopsin in photoreceptors. (a-c) Confocal images of control fly eyes (Rh1-GAL4) and eyes expressing htau^WT^ (d-f, RH1::htau^WT^). Newly eclosed adult eyes are stained with anti-rhodopsin (4C5, green) and rhodamine-phalloidin (red). Aberrant accumulation of rhodopsin staining in htau^WT^ eye (e) is evident as compared to control (b). Scale bar, 100 µm. (TIFF 8084 kb)
