# Supplementary figures and images for: Loss of vesicular dopamine release precedes tauopathy in degenerative dopaminergic neurons in a Drosophila model expressing human tau
Source: Acta Neuropathol. 2013 Mar 15;125(5):711–25. doi: 10.1007/s00401-013-1105-x (PMC3631315; doi:10.1007/s00401-013-1105-x)

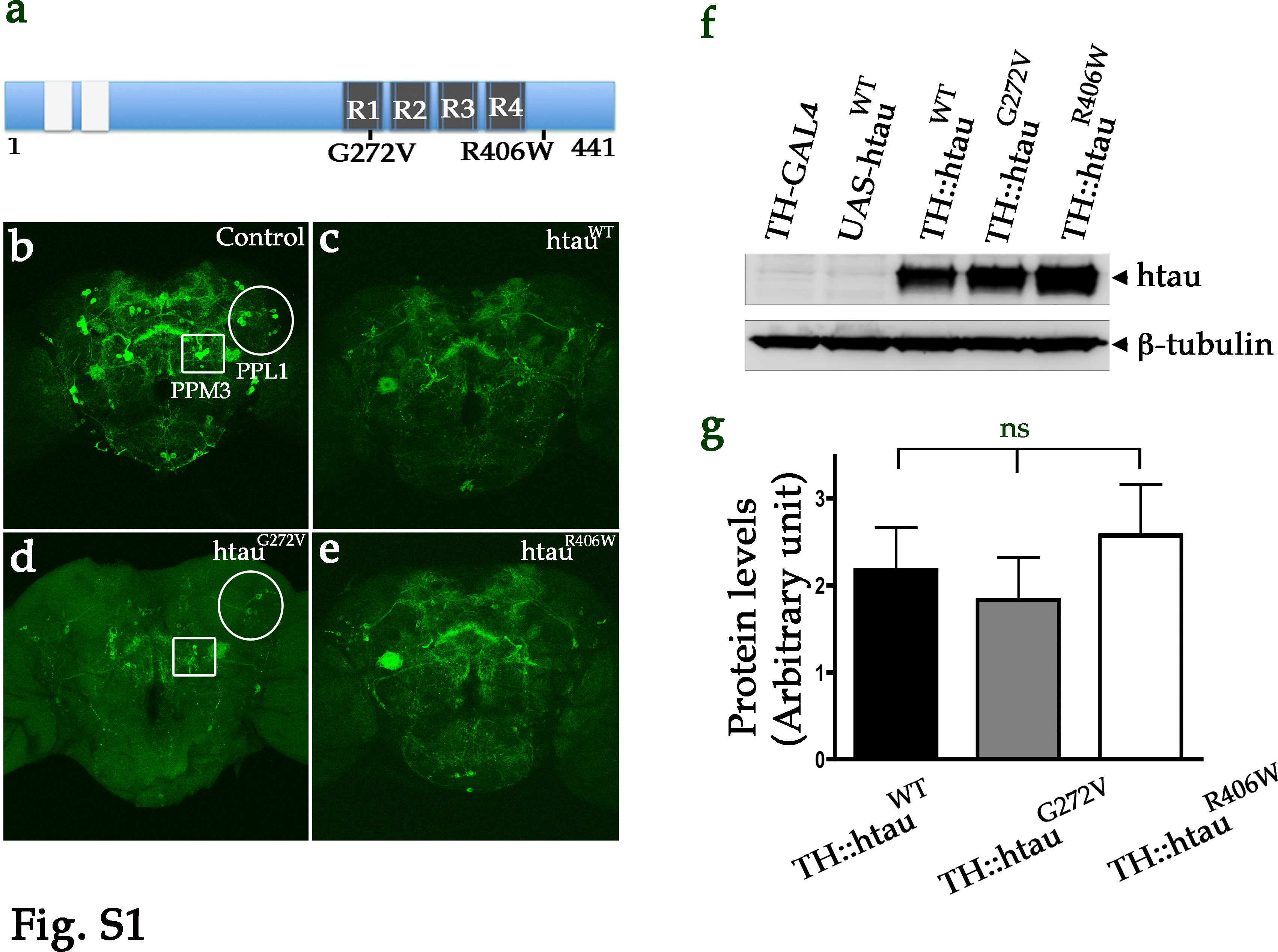

Supplement: Supplementary file 1 — Supplementary material 1 Expression of htauG272V and htauR406W cause DA neuron degeneration similar to htauWT. Related to Fig 1. (a) A depicted human tau isoform with 441 amino acids. The N domains are shown in white boxes and the C-terminal microtubule binding repeats are shown in black boxes with labeled as R1-R4. Two FTDP-17 associated mutations, G272V and R406W, are indicated. (b-e) Representative confocal images show mCD8-GFP-marked DA neurons in four-week-old control fly brain (b, TH::mCD8-GFP), and age-matched brains from flies expressing htauWT (c, TH:: htauWT, mCD8-GFP), htauG272V (d, TH:: htauG272V, mCD8-GFP), and htauR406W (e, TH:: htauR406W, mCD8-GFP). Two clusters of DA neurons, PPL1 (circles) and PPM3 (squares), are indicated. (f) Representative western blot shows protein levels of htauWT, htauG272V, and htauR406W that expressing in DA neurons. No human tau proteins can be detected in TH::mCD8-GFP and UAS-htauWT controls. β-Tubulin serves as a loading control. (g) Quantification of four independent western blots. Values shown represent Mean ± SEM; one-way ANOVA, P = 0.6185; ns, not significant. (TIFF 3756 kb) [file 401_2013_1105_MOESM1_ESM.tif]

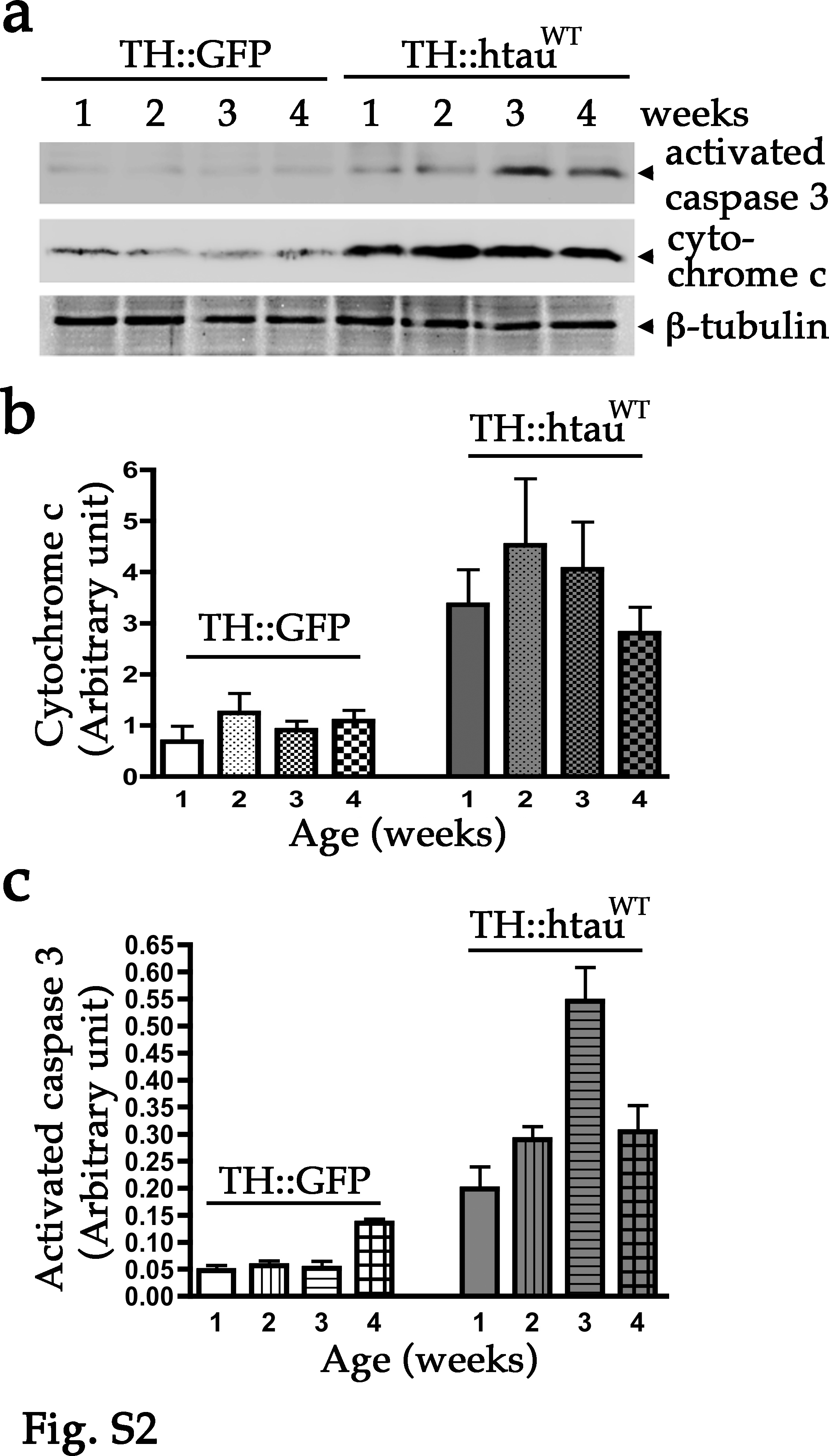

Supplement: Supplementary file 2 — Supplementary material 2 Expression of htauWT activates cell death signaling. (a) Representative immunoblot shows increased cytochrome c and activated caspase 3-like caspase in brains with DA neurons that expressing htauWT (TH::htauWT) compared to age-matched control (TH::GFP). The decrease of caspase 3-like signal at the fourth week is likely influenced by DA neuron loss by this age. Anti-ß-tubulin serves as a loading control. Quantification from four independent immunoblots shows expression levels of cytochrome c (b) and activated caspase 3 (c) represented as relative expression. Values shown represent Mean ± SEM. (TIFF 923 kb) [file 401_2013_1105_MOESM2_ESM.tif]

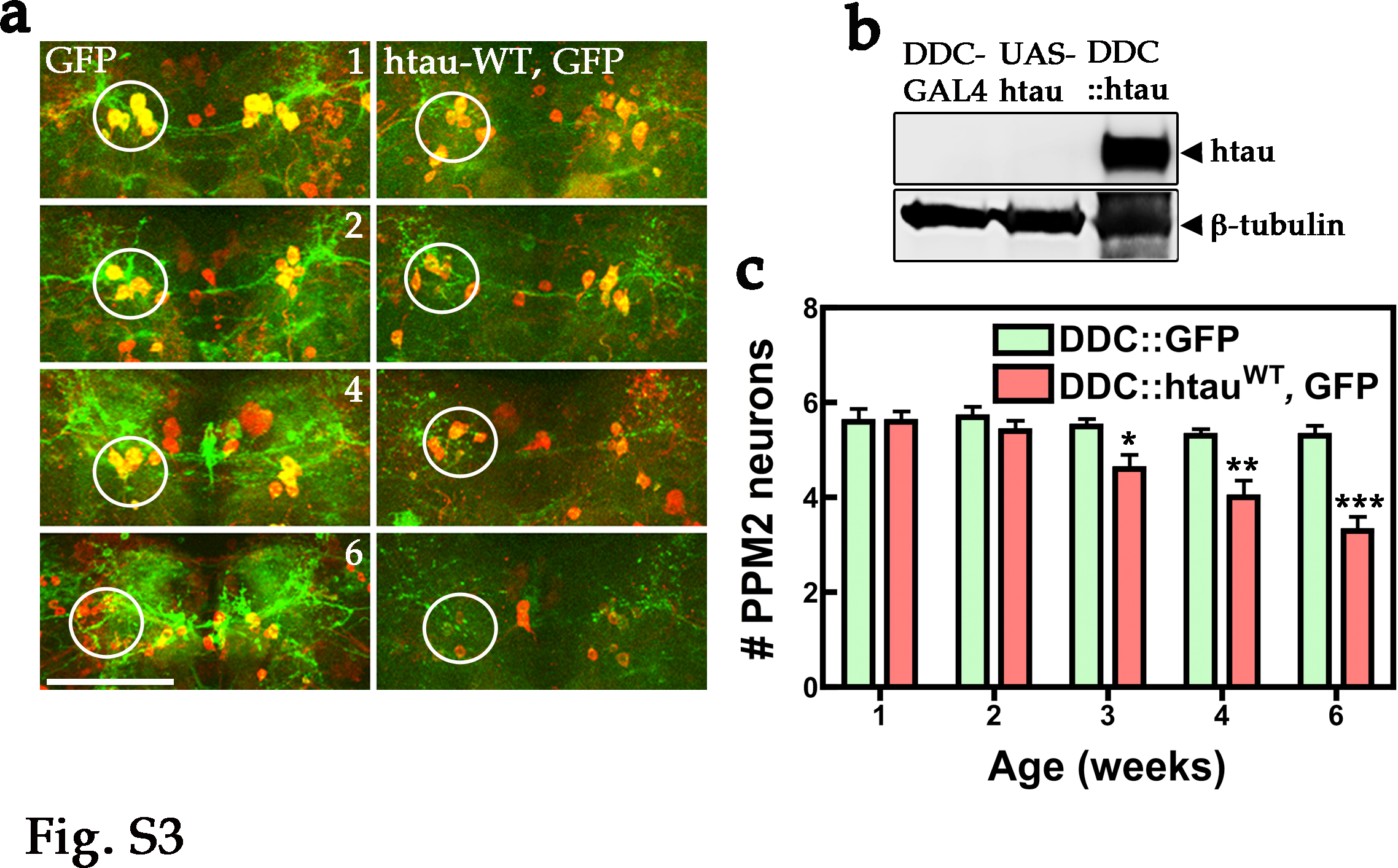

Supplement: Supplementary file 3 — Supplementary material 3 Expression of htauWT in DA and 5HT neurons also induced DA neuron degeneration. Related to Fig 1. (a) Representative confocal images of control fly brains (left panels, DDC::mCD8-GFP) and brains expressing htauWT (right panels, DDC::htauWT, mCD8-GFP) at different ages (weeks numbered in the upper right corner of the left panels) stained with anti-tyrosine hydroxylase (anti-TH, red). PPM2 clusters of DA neurons (circles) are marked with both anti-TH and GFP (yellow). Scale bar, 50 µm. (b) Immunoblotting of a polyclonal Tau antibody (against the C-terminal amino acids 243-441) detects a 75 kDa band in DDC:: htauWT, but not in DDC-GAL4 or UAS-htauWT controls. Anti-tubulin served as a loading control. (c) Quantitative analysis shows the number of DA neurons in PPM2 clusters in htauWT (pink) and control (green) at indicated ages. Values shown represent Mean ± SEM (unpaired t-test, *P < 0.01; ** P < 0.001; ***P < 0.0001, n = 10). (TIFF 2561 kb) [file 401_2013_1105_MOESM3_ESM.tif]

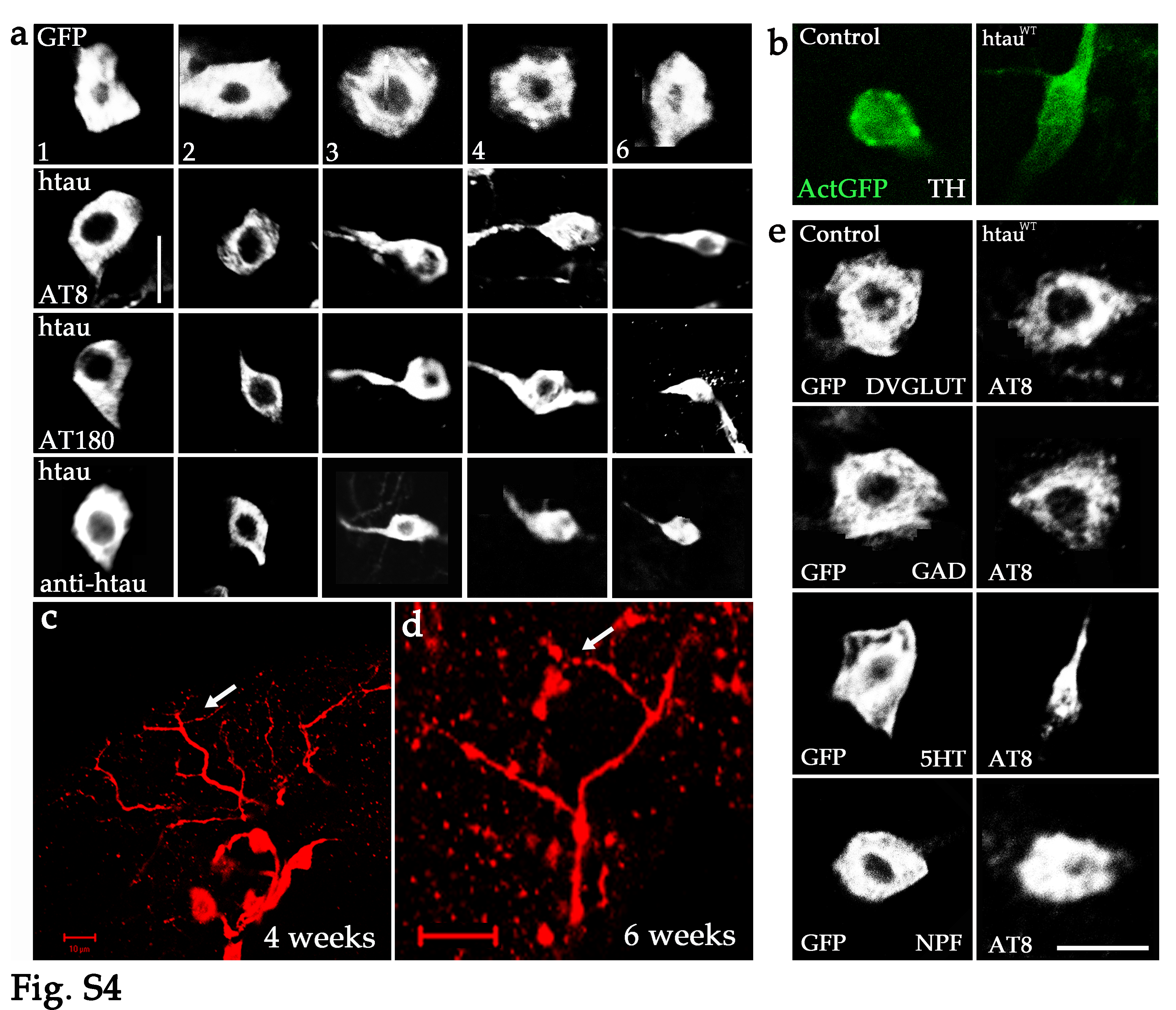

Supplement: Supplementary file 4 — Supplementary material 4 The time course of developing tangle-like pathology in the degenerating DA neurons and the effect of htauWT on other types of neurons. Related to Figure 4. (a) Confocal images focusing at a single DA neuron from control (TH::mCD8-GFP) and htauWT expressing (TH-GAL4:: UAS-htauWT, mCD8-GFP) brains at indicated ages (in week indicated at the lower left corner of row). GFP-marked DA neuron in control brains shows comparable soma size at different ages (a), while DA neuron expressing htauWT reveals progressive reduction of soma size as well as the formation of tangle-like pathology extended from the soma begin at approximate 3 weeks of age, featured by AD-like hyperphosphorylated tau antibodies AT8 and AT180 staining and a polyclonal Tau antibody (a). (b) Confocal images of a single DA neuron marked by actin-GFP reveal edges of the soma are associated with actin rich puncta in the control brain (TH::actin-GFP), but are absent of these actin rich puncta on the surface of the soma in the htauWT expressing brain. Confocal images of AT8 immunostaining show the fragmented axonal branches (arrows) at 4 weeks (c) and 6 weeks (d) htauWT expressing DA neurons. (e) Other types of neurons expressing htauWT show comparable soma size and shape (right panels) as compared to normal glutamatergic (top row), GABAergic (second row from top) and NPF (bottom row) neurons (left panels). In contrast, 5HT neurons expressing htauWT show reduction in soma size and morphological change, similar to that of DA neurons in 6-week-old flies. Scale bar, 10 µm. (TIFF 3941 kb) [file 401_2013_1105_MOESM4_ESM.tif]

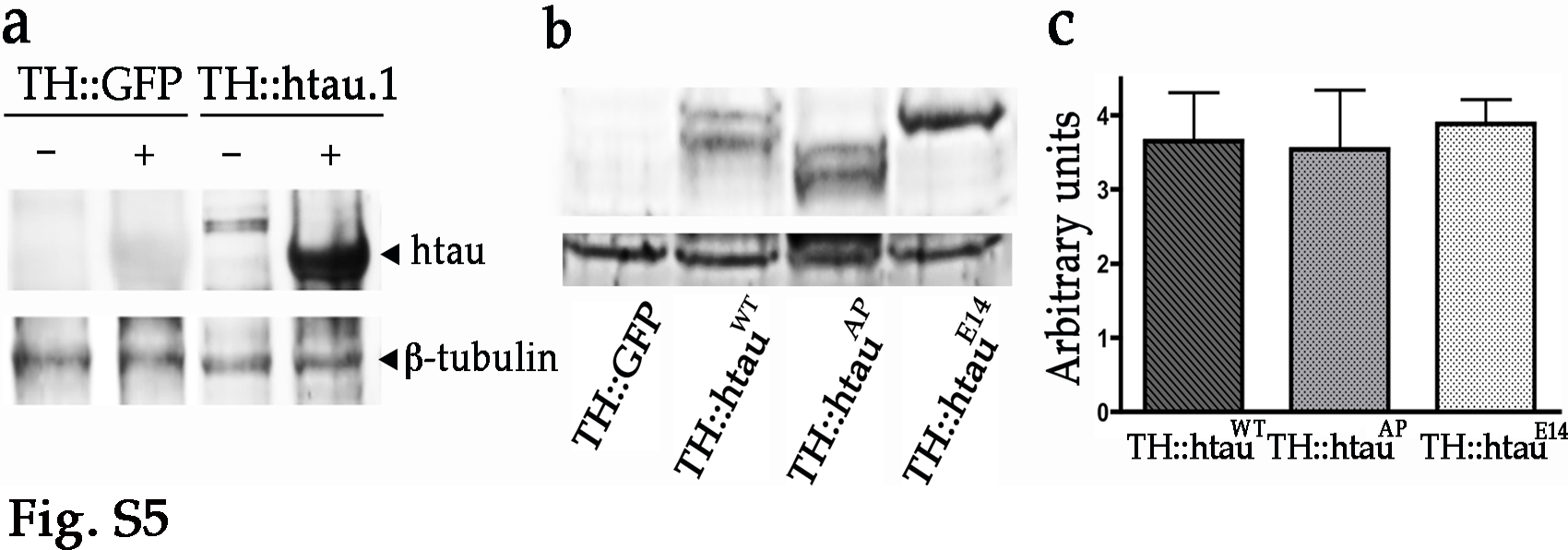

Supplement: Supplementary file 5 — Supplementary material 5 Analysis of phosphorylation states of htauWT proteins and protein levels of three human tau alleles: htauAP, htauWT, and htauE14 in DA neurons. (a) Representative western blot shows expression of htauWT protein in DA neurons were phosphorylated (-) and the migration in SDS-PAGE is slower than those htauWT proteins treated with alkaline phosphatase (+). No human tau proteins can be detected in control TH::GFP. β-Tubulin serves as a loading control. (b) Representative western blot shows protein levels of three htau alleles in DA neurons: htauWT (TH::mCD8-GFP, htauWT), htauAP (TH::mCD8-GFP, htauAP), and htauE14 (TH::mCD8-GFP, htauE14). β-Tubulin serves as a loading control. (c) Quantification of three independent western blots. Values shown represent Mean ± SEM; one-way ANOVA, P = 0.925; n = 3). (TIFF 474 kb) [file 401_2013_1105_MOESM5_ESM.tif]

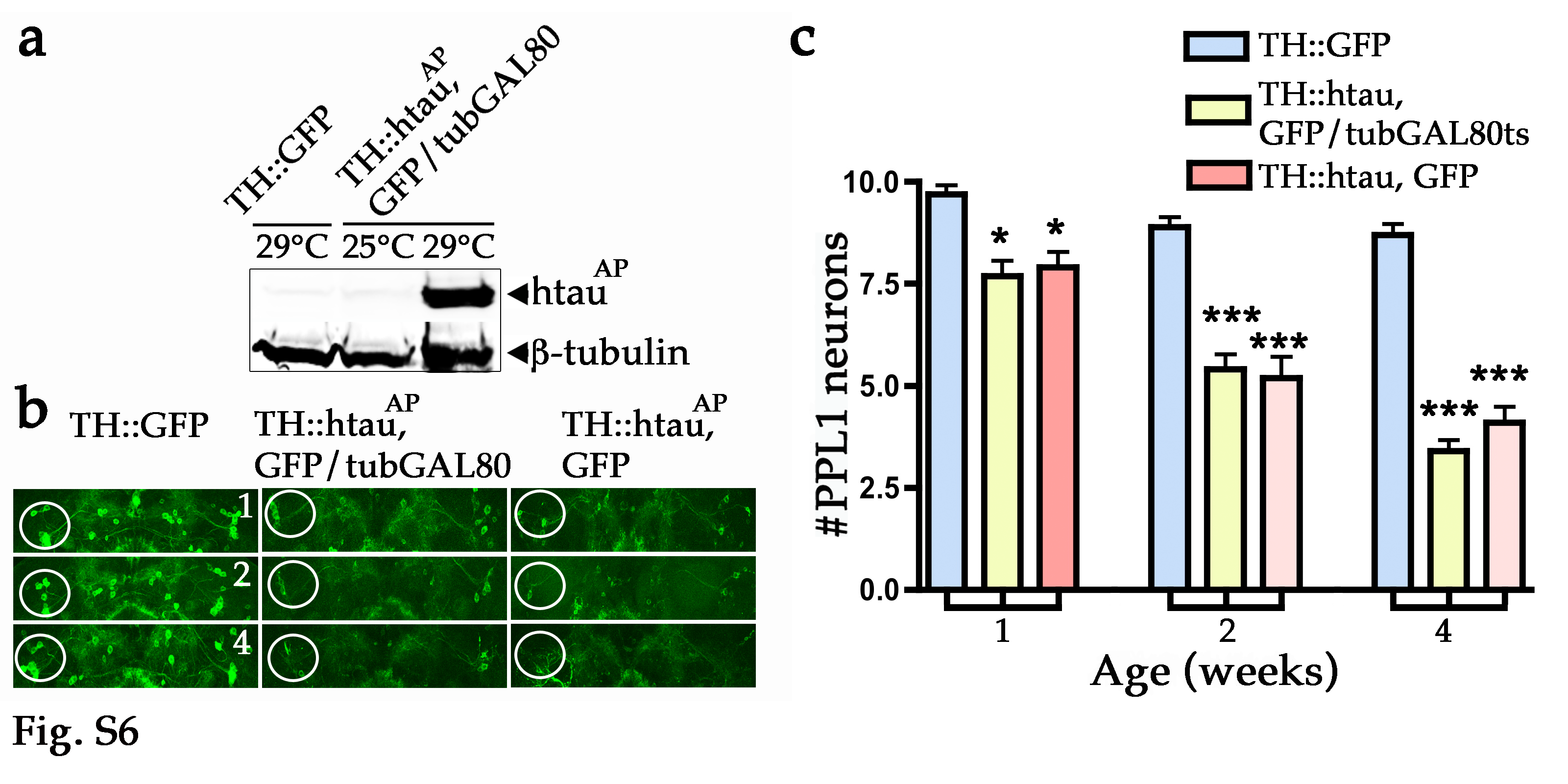

Supplement: Supplementary file 6 — Supplementary material 6 Adult onset of htauAP expression also produces severe age-dependent neurodegeneration. Related to Figure 5. (a) Representative western blot shows conditional expression of htauAP. At room temperature (25°C), tub-GAL80ts represses transgene expression, thus htauAP is silenced (TH::htauAP,mCD8-GFP/tubGAL80ts at 25°C); whereas at 29°C, tub-GAL80ts fails to repress transgene expression, thus the expression of htauAP transgene is detected (TH::htauAP, mCD8-GFP/tubGAL80ts at 29°C). No htauAP is detected in control (TH::mCD8-GFP). (b) Representative confocal images of PPL1 groups of DA neurons marked with mCD8-GFP (TH::GFP) in the control, conditional expression of htauAP in adult flies (TH::htauAP, mCD8-GFP/tubGAL80ts), or developmental expression of htauAP (TH::htauAP, mCD8-GFP) at indicated ages. Circles indicate the PPL1 clusters. (c) Quantitative analysis presents DA neuron numbers in PPL1 cluster at different ages (weeks) from control and htauAP with indicted expression regimes. TH:: mCD8-GFP (blue); TH::htauAP, mCD8-GFP/tubGAL80ts (yellow); TH::htauAP, mCD8-GFP (pink). Values shown represent Mean ± SEM (unpaired t test compares individual tau allele to the control in the age-matched groups; *P < 0.01; ***P < 0.0001, n = 12). (TIFF 1482 kb) [file 401_2013_1105_MOESM6_ESM.tif]

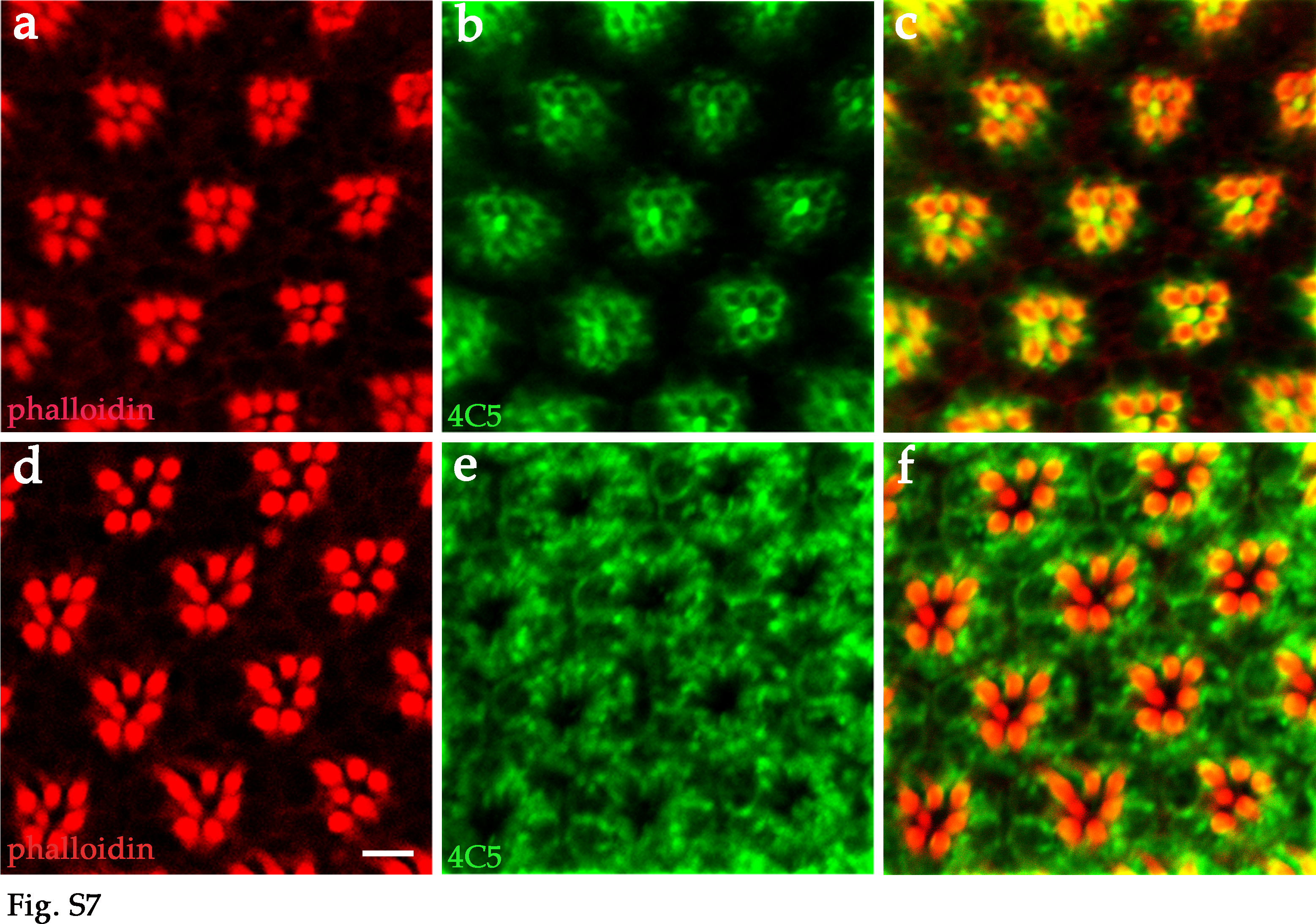

Supplement: Supplementary file 7 — Supplementary material 7 Expression of htauWT causes the mislocalization of rhodopsin in photoreceptors. (a-c) Confocal images of control fly eyes (Rh1-GAL4) and eyes expressing htauWT (d-f, RH1::htauWT). Newly eclosed adult eyes are stained with anti-rhodopsin (4C5, green) and rhodamine-phalloidin (red). Aberrant accumulation of rhodopsin staining in htauWT eye (e) is evident as compared to control (b). Scale bar, 100 µm. (TIFF 8084 kb) [file 401_2013_1105_MOESM7_ESM.tif]
